# A Systematic Review of the Prevalence and Pattern of Imaging Defined Post-TB Lung Disease

**DOI:** 10.1371/journal.pone.0161176

**Published:** 2016-08-12

**Authors:** Jamilah Meghji, Hope Simpson, S. Bertel Squire, Kevin Mortimer

**Affiliations:** 1 Malawi-Liverpool-Wellcome Clinical Research Programme, Blantyre, Malawi; 2 Department of Clinical Sciences, Liverpool School of Tropical Medicine, Liverpool, United Kingdom; University of Otago, NEW ZEALAND

## Abstract

**Background:**

Tuberculosis is an important risk factor for chronic respiratory disease in resource poor settings. The persistence of abnormal spirometry and symptoms after treatment are well described, but the structural abnormalities underlying these changes remain poorly defined, limiting our ability to phenotype post-TB lung disease in to meaningful categories for clinical management, prognostication, and ongoing research. The relationship between post-TB lung damage and patient-centred outcomes including functional impairment, respiratory symptoms, and health related quality of life also remains unclear.

**Methods:**

We performed a systematic literature review to determine the prevalence and pattern of imaging-defined lung pathology in adults after medical treatment for pleural, miliary, or pulmonary TB disease. Data were collected on study characteristics, and the modality, timing, and findings of thoracic imaging. The proportion of studies relating imaging findings to spirometry results and patient morbidity was recorded. Study quality was assessed using a modified Newcastle-Ottowa score. (Prospero Registration number CRD42015027958)

**Results:**

We identified 37 eligible studies. The principle features seen on CXR were cavitation (8.3–83.7%), bronchiectasis (4.3–11.2%), and fibrosis (25.0–70.4%), but prevalence was highly variable. CT imaging identified a wider range of residual abnormalities than CXR, including nodules (25.0–55.8%), consolidation (3.7–19.2%), and emphysema (15.0–45.0%). The prevalence of cavitation was generally lower (7.4–34.6%) and bronchiectasis higher (35.0–86.0%) on CT vs. CXR imaging. A paucity of prospective data, and data from HIV-infected adults and sub-Saharan Africa (sSA) was noted. Few studies related structural damage to physiological impairment, respiratory symptoms, or patient morbidity.

**Conclusions:**

Post-TB structural lung pathology is common. Prospective data are required to determine the evolution of this lung damage and its associated morbidity over time. Further data are required from HIV-infected groups and those living in sSA.

## Introduction

Chronic respiratory diseases (CRDs) are the fourth leading cause of non-communicable disease (NCD) deaths globally, and pose a particular challenge to low and middle-income countries (LMICs) where risk factors for respiratory damage including poverty-related in-utero and early childhood exposures[1], exposure to acute respiratory infections[2], indoor biomass fuel exposure[3, 4], a rising prevalence of smoking[5], chronic HIV-infection[6], and pulmonary tuberculosis (PTB) intersect. A high prevalence of CRDs has been demonstrated in LMICs, and these are expected to have a substantial impact on population health and health-services in the coming years[7–10]. An improved understanding of the nature of CRDs in LMICs, their natural history, and associated morbidity and mortality is required if we are to design appropriate prevention, diagnostic, and management strategies[11, 12].

Pulmonary TB remains an important cause of chronic respiratory impairment in LMICs. 5.2 million incident cases of PTB were reported globally in 2014[13], and the presence of long term respiratory sequelae following PTB treatment is well established [14, 15]. Pulmonary granuloma formation, tissue necrosis and liquefaction, and aberrant healing responses are known features of TB disease[16, 17]. The persistence of abnormal airway physiology after treatment has been documented in large population-based cross-sectional studies which show 1.37–2.94 higher odds of fixed airways obstruction in those with a history of PTB, compared to those without[15, 18–21]. Previous TB has also been associated with chronic respiratory symptoms in LMICS: previous TB was the strongest predictor of chronic bronchitis within the 1996 South African Demographic & Health Survey [22] and the odds of a medical diagnosis of bronchiectasis were over 3-fold higher in those who had a history of TB, compared to those who had not, in a population based study of 10,811 adults in China [23].

Whilst the evidence for abnormal spirometry and symptoms following PTB disease in LMICS is clear, imaging of patients completing PTB treatment is not routinely performed, and our understanding of the associated patterns of structural lung pathology remains limited. Without these imaging data, we are not yet able to accurately phenotype patterns of post-TB lung disease in to the meaningful categories required for clinical management, prognostication, and ongoing research into the risk factors for post-TB lung damage [24, 25]. In addition, information on the morbidity and mortality associated with post-TB lung damage remains limited, but would be timely given the post-2015 TB agenda, which recognises the need for TB services to mitigate the long-term detrimental impact of TB disease on patients’ lives and livelihoods [26].

In this review we seek to improve our understanding of the nature and impact of post-TB lung disease. We examine the literature on imaging defined structural post-TB lung damage, and its relationship to patient-centred outcomes including functional impairment, respiratory symptoms, and health-related quality of life. We have included studies using both plain chest radiographs (CXR) and more detailed computerised tomography (CT) imaging in our review: CXR is widely available for use in TB screening and diagnosis and may be of programmatic use in the diagnosis and management of post-TB lung damage, but CT provides a higher level of detail and may provide more information on the true nature of this damage.

## Methods

A protocol driven literature search was performed following PRISMA guidelines to identify studies in which consecutive participants with pleural, miliary, or pulmonary TB were recruited, where CXR or CT was performed after the completion of a full medical TB treatment regimen, and where the prevalence of abnormal imaging or the severity of residual structural lung damage was reported (S1 File). Cohort studies, cross-sectional studies and randomised control trials (RCTs) were eligible for inclusion. There were no limits on publication date. Only studies published in English were included.

Literature searches were conducted in Medline, Pubmed, Scopus, Web of Science, and the Cochrane Library (July 2016) (Table 1). Reference lists from published reviews and reference and citation lists of papers meeting inclusion criteria were reviewed to identify additional articles.

**Table 1:** Template for literature search: Pulmonary, pleural or military tuberculosis AND [CXR imaging OR CT imaging].

Criteria	Search terms
Pulmonary, pleural, or miliary tuberculosis	“Tuberculosis, pulmonary”[Mesh] OR “tuberculosis, miliary[Mesh] OR “tuberculosis, pleural”[Mesh] OR "pulmonary TB" OR "pulmonary tuberculosis"
CXR Imaging	“thoracic radiography"[MeSH] OR “chest x-ray" OR "chest radiograph" OR "CXR"
CT imaging	“computed tomography”[MeSH] OR "CT" OR "comput* tomography"

The title and abstract of all identified studies were screened by two independent reviewers (JM and HS). Full text review was performed on all selected articles. Studies restricted to paediatric populations, in whom PTB has a varied presentation, or patients with non-HIV related immunosuppression (chemotherapy, malignancy etc.) where imaging was likely to be affected by comorbidities, were excluded. Data from the control arms of trials of adjuvant immunomodulatory therapies, in which patients received medical TB treatment only, were included.

A standardized data extraction form was used by 2 authors (JM and HS) to determine the primary outcome of interest, which was the prevalence of abnormal imaging after TB treatment. Information was collected on the patterns of imaging pathology, study characteristics, participant characteristics, treatment regimens, and the modality and timing of thoracic imaging. We recorded the proportion of studies that presented a measure of association between imaging findings and other clinical parameters including spirometry, functional capacity, respiratory symptoms, or health-related quality of life. Disagreements in study selection and data extraction were resolved by discussion. Subgroup analyses were conducted to explore the effect of different manifestations of disease (pleural vs. pulmonary), imaging modality (CXR vs. CT), and multidrug-resistant (MDR) disease on the primary outcome. A narrative analysis was conducted.

Study quality was determined using a modified version of the Newcastle-Ottowa score[27], which included assessment of selection bias, adequacy of follow-up, the accuracy with which baseline TB disease and treatment completion were determined, the quality and standardisation of imaging interpretation, and the exclusion of those with structural lung disease preceding TB-disease. A maximum score of 5 was possible for cohort studies, and 4 for cross-sectional studies where no follow-up was required.

## Results

**Fig 1:**
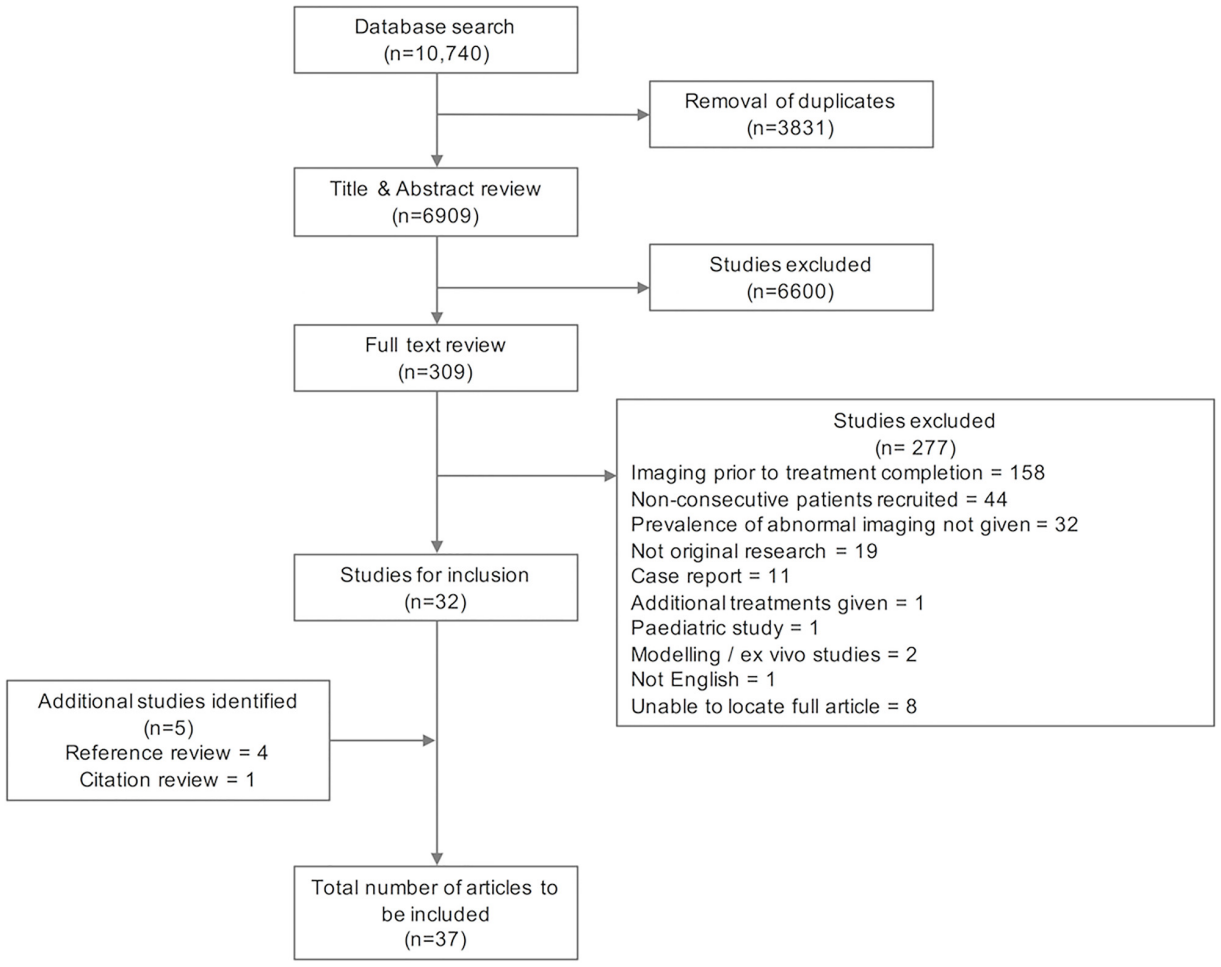
PRISMA flow chart.

We identified 10,740 articles, with 6909 articles remaining after removal of duplicates. Title and abstract review identified 309 articles for full text review, of which 277 were excluded for reasons including non-consecutive patient recruitment, imaging prior to treatment completion, and failure to report the absolute prevalence or severity of residual lung damage. Reference and citation searches identified 5 further articles for inclusion, giving a total of 37 articles (Fig 1).

These papers covered the time period 1973–2015. They included 16 prospective cohort studies with patients recruited at TB diagnosis and imaged upon treatment completion, and one prospective cohort study where imaging was performed 1 year post treatment completion[28]. There were 7 cross-sectional studies, 5 of which performed imaging at various time points after treatment completion, and 7 retrospective cohort studies, which performed imaging upon treatment completion. Data from 6 RCTs were included: data from both study arms were included for two treatment regimen trials [29, 30] and a study investigating the effect of Vitamin-D and L-arginine supplementation[31], but data from the control arms only were included from trials investigating prednisolone use[32, 33], and a trial of *M*. *vaccae* immunomodulation[34]. All RCTs performed imaging at treatment completion, and 1 also performed serial imaging 6-months later[32]. Only seven studies used CT imaging to describe structural pathology. Five of these were conducted in the Americas, and all performed imaging at treatment completion. Only one study of pleural disease used both CXR and CT imaging[32].

The total number of patients in all included studies was 4870, with disaggregated data available for 76 HIV-infected individuals. The median number of participants per study was 131 (range 13–1080). Studies were conducted in the Americas (n = 11), South East Asia (n = 9), Europe (n = 6), the Western Pacific region (n = 6), and Africa (n = 2). Both of the studies conducted in Africa were from South Africa and focused on either pleural[32] or MDR disease[35]. 26 studies focused on PTB, 1 on the sequelae of miliary TB, 9 were restricted to pleural TB, and 1 included patients with pulmonary, pleural or mediastinal TB[36]. The marked heterogeneity between studies made meta-analysis of their findings inappropriate.

Few studies specified the pattern of drug sensitivity (12/37), or previous episodes of TB (10/37). Two studies of residual lung damage in patients receiving TB retreatment were included, one of which was restricted to MDR patients[34, 37]. Two additional studies of patients with MDR disease but unspecified histories of TB treatment were identified[35, 38]. The treatment regimens used varied widely; only 7 studies specified use of the gold standard short-course treatment regimen, and only 2 out of 3 studies of MDR disease specified the use of national treatment guidelines[35, 37].

### Pulmonary disease

Of the 27 studies reporting the sequelae of pulmonary and miliary TB, prevalence estimates for radiographic pathology were given in 17 CXR (Table 2) and 5 CT studies (Table 3), and varied widely. Twelve CXR studies reported the prevalence of cavitation (8.3–83.7%), 3 reported fibrosis (prevalence 25.0–70.4%) and 4 reported bronchiectasis (prevalence 4.3–11.2%). The CT-based studies generally reported a lower prevalence of cavitation (7.4–34.6%), and a higher prevalence of bronchiectasis (35.0–86.0%) and fibrosis (70.0–92.6%), than studies using CXR imaging. A more diverse range of pathologies was noted on CT imaging: pleural thickening was reported in 3 studies (prevalence 0.1–50.0%, n = 99), features potentially suggestive of ongoing inflammation such as nodules were seen in all 5 studies (prevalence 25.9–55.8%, n = 193), consolidation was reported in 4 studies (3.7–19.2%, n = 119), emphysema was seen in in 2 studies (prevalence 15.4–45.0%, n = 72), and mosaicism was documented in 1 study (prevalence 70%, n = 20). A broad range of severity scores were used to quantify residual damage in 13 studies of PTB sequelae, only one of which was validated for scoring post-TB damage rather than active PTB disease[39] (Table 4).

**Table 2:** Studies reporting prevalence of imaging patterns on CXR imaging following treatment for thoracic tuberculosis.

Timing of imaging	Author, Year	Country	Study design	TB pattern	Participant HIV status	Treatment episode	Drug sensitivity	Number of participants	Prevalence of pathology (%)	Quality score
On completion of TB Treatment	Yu, 1995[40]	Taiwan	Prospective cohort	Pulmonary	Negative	Not specified	Mixed	22	Abnormal imaging: 13.6%	3/5
	Al Hajjaj, 2000 [41]	Saudi Arabia	Prospective cohort	Pulmonary	Not specified	Not specified	Not specified	1080	Abnormal imaging: 65.9%, Cavitation: 15.0%, Pleural thickening 6.9%, Lung destruction 52.4%	3/5
	de Valliere, 2004[35]	South Africa	Prospective cohort	Pulmonary	Mixed	Not specified	MDR	33	Abnormal imaging: 93.9–100%, Cavitation: 51.5%–69.7%	2/5
	Buyokoglan, 2007 [42]	Turkey	Prospective cohort	Pulmonary	Negative	Not specified	Not specified	25	Cavitation: 28.0%	3/5
	Swaminathan, 2007 [43]	India	Prospective cohort	Miliary	Positive	Not specified	Not specified	31	Abnormal imaging: 22.6%. Lung destruction: 3.2%	3/5
	Angthong, 2011 [44]	Thailand	Prospective cohort	Pulmonary	Mixed—data disaggregated	First episode	Not specified	98 HIV+	Abnormal imaging: 84.7%, Cavitation: 11.2%, Fibrosis: 70.4%, Bronchiectasis: 11.2%	4/5
								12 HIV-	Abnormal imaging: 41.7%, Cavitation: 8.3%, Fibrosis: 25.0%, Bronchiectasis: 8.3%	
	Small, 1994 [45]	America	Retrospective cohort	Pulmonary	Positive	Not specified	Not specified	13	Abnormal imaging: 23.1%	3/5
	Menon, 2015 [36]	India	Retrospective cohort	Pulmonary, pleural, mediastinal	Not specified	First episode	Not specified	441	Abnormal imaging: 40.4, Cavitation: 21.4%, Pleural thickening: 21.2%, Fibrosis: 38.7%, Bronchiectasis: 4.3%, Mediastinal lesions: 23.6%	2/5
	Kallan, 1988 [46]	India	Cross sectional	Pulmonary	Not specified	Not specified	Not specified	119	Abnormal imaging: 100.0%, Cavitation: 42.0%, Bronchiectasis: 7.6%	1/4
	Anonymous, 1973 [29]	India	RCT–TB treatment regimens*	Pulmonary	Not specified	Not specified	Not specified	173	Cavitation: 49.7%	4/5
	Hamilton, 2008 [30]	America	RC –TB treatment regimens*	Pulmonary	Negative	Not specified	Fully sensitive	834	Cavitation: 23.3%	4/5
	Kenangalem, 2013 [31]	Indonesia	RCT–additional Vit D / L-arginine†	Pulmonary	Mixed	First episode	Mixed	77	Cavitation: 18.2%	2/5
On completion and at 6m	Corlan, 1997 [34]	Romania	RCT–additional M.vaccae†	Pulmonary	Retreatment	Mixed	Mixed	43 with imaging on completion	Cavitation: 83.7%	3/5
								32 with imaging at 6 months	Cavitation: 68.8%	
6–63 months post completion	Singla, 2009 [38]	India	Cross sectional	Pulmonary	Negative	Not specified	MDR	45	Abnormal imaging: 97.8%, Cavitation: 53.3%	1/4
14–18 years post completion	Banu Rekha, 2009 [47]	India	Cross sectional	Pulmonary	Not specified	First episode	Not specified	198	Abnormal imaging: 85.9%	1/4
5 years post completion	Lisha, 2012 [48]	India	Cross sectional	Pulmonary	Not specified	Mixed	Mixed	224	Abnormal imaging: 65.6%	2/4
0–252 months post completion	Baez-saldana, 2013 [39]	Mexico	Cross sectional	Pulmonary	Mixed	Not specified	Not specified	127	Abnormal imaging: 96.9%	2/4

*Data included from both arms

^†^Data included from placebo arms only

**Table 3:** Studies reporting prevalence of imaging patterns on CT imaging on completion of treatment for pulmonary tuberculosis.

Author, Year	Country	Study design	Participant HIV status	Treatment episode	Drug sensitivity	Number of participants	Prevalence of pathology (%)	Quality score
Poey, 1997[49]	Martinique	Prospective cohort	Negative	Not specified	Not specified	27	Cavitation: 7.4%, Bronchiectasis: 85.2%, Fibrosis: 92.6%, Pleural thickening: 4.8%, Nodules: 25.9%, Consolidation: 3.7%, Ground glass pattern: 7.4%, Reticulation: 44.4%,	3/5
Long, 1998 [50]	Canada	Prospective cohort	Negative	Not specified	Fully sensitive	20	Bronchiectasis: 50.0%, Fibrosis: 80.0%, Pleural thickening: 0.1%, Nodules: 55.0%, Consolidation: 15.0%, Emphysema/Bullae: 45.0%, Mosaicism: 70.0%	3/5
Bombarda, 2003 [51]	Brazil	Prospective cohort	Negative	Not specified	Not specified	20	Cavitation: 30.0%, Bronchiectasis: 35.0%, Fibrotic bands: 70.0%, Nodules: 55.0%, Consolidation: 15.0%, Mass lesions: 45%	3/5
Lee, 2008 [52]	Taiwan	Prospective cohort	Negative	First episode	Fully sensitive	52	Cavitation: 34.6%, Bronchiectasis: 44.2%, Fibrosis: 92.3%, Pleural thickening: 50.0%, Nodules: 55.8%, Consolidation: 19.2%, Emphysema/Bullae: 15.4%, Mass lesions: 7.7%, Ground glass pattern: 1.9%, Parenchymal calcification: 11.5%	1/5
Rufino, 2015[53]	Brazil	Prospective cohort	Not specified	Not specified	Not specified	74	Cavitation: 16%, Bronchiectasis: 86%, Nodules: 48%, Parenchymal opacities: 25%, Parenchymal calcifications: 47%, Architectural distortion: 91%	1/5

**Table 4:** Studies reporting severity scores of residual changes on CXR imaging performed following treatment for pulmonary TB.

Author	Country	Study design	n	Participant HIV status	Treatment episode	Drug sensitivity	Timing of imaging	Source of severity score	Severity score description	Findings	Quality score
de Valliere, 2004 [35]	South Africa	Prospective cohort	33	Mixed	Not specified	MDR	On completion	Not specified	CXR split into 6 zones. Involvement of each zone scored 0–3. Total score 18.	Mean score 6.5/18	2/5
Ralph, 2010 [54]	Indonesia	Prospective cohort	152	Mixed	Not specified	Mixed	On completion	Ralph 2010—diagnostic CXR scoring system	% lung affected + 40 if cavitation seen. Total score 140.	Median score 10/140, Range 0–115	3/5
Wang, 2010 [28]	Taiwan	Prospective cohort	98	Negative	Not specified	Not specified	1 year post completion	Not specified	Minimal / Moderate / Advanced fibrosis*	60.2% Minimal; 14.3% Moderate; 25.5% Advanced	3/5
Chen, 2011 [55]	Taiwan	Prospective cohort	51	Negative	Not specified	Not specified	On completion	McAdams & Erasmus 1995—active TB CXR scoring system	Minimal / Extensive†	31.4% Extensive	3/5
Menon, 2015[36]‡	India	Retrospective cohort	441	Not specified	First episode	Fully sensitive	On completion	1969 National TB associate of the USA—diagnostic CXR scoring system	Minimal / Moderate / Moderately advanced / Far advanced	55.7% Minimal; 22.8% Moderate; 15.2% Moderately advanced; 6% Advanced	2/5
How, 2014 [56]	Malaysia	Retrospective cohort	156	Mixed	Mixed	Not specified	On completion	1961 National TB association USA -diagnostic CXR scoring system [57]	Minimal / Moderate / Advanced disease^¶^	26.2% Minimal; 60.8% Moderate; 13% Advanced	2/5
Singla, 2009 [38]	India	Cross sectional	45	Negative	Not specified	MDR	6-63m post completion	1961 National TB association USA -diagnostic CXR scoring system[57]	Minimal / Moderate / Advanced disease^¶^	35.6% Minimal; 22.2% Moderate; 40.0% Advanced	1/4
Lisha, 2012 [48]	India	Cross sectional	224	Not specified	Mixed	Mixed	5 years post completion	1961 National TB association USA -diagnostic CXR scoring system [57]	Minimal / Moderate / Advanced disease^¶^	34.3% Minimal; 13.4% Moderate; 4.5% Advanced	2/4
Banu Rekha, 2009 [47]	India	Cross sectional	198	Not specified	First episode	Not specified	14–18 years post completion	Not specified	CXR divided into 6 zones. Number of zones involved counted.	35.9% ≤2 zones; 50% >2 zones	1/4
Godoy, 2012 [37]	Brazil	Cross sectional	18	Negative	Retreatment	MDR	On completion	Wilcox & Ferguson 1989—diagnostic CXR scoring system [58]	Grade I—III^#^	61.1% Grade I; 22.2% Grade II; 16.7% Grade III	2/4
Baez-saldana, 2013 [39]	Mexico	Cross sectional	127	Mixed	Not specified	Not specified	0–252 months post completion	Created by authors for grading post-TB CXR changes & validated in study	CXR split into 4 quadrants. Involvement of each one scored 0–5. Total score 20.	Mean score 6.46/20, standard deviation 4.14	2/4
de la Mora, 2015 [59]	Mexico	Cross sectional	70**	Not specified	Mixed	Mixed	Post completion. With CAO: 2.7+/- 4.3 yrs. Without CAO: 2.3 +/- 2.1 yrs	Not specified	Number of lung quadrants with fibrocavitatory changes. Total number of cavities	With CAO: 1.8 +/-0.8 affected quadrants, 1.4 +/- 0.8 cavities. Without CAO: 1.3 +/- 0.6 affected quadrants, 0.5 +/-0.7 cavities	1/4
Kenangalem, 2013 [31]	Indonesia	RCT—Vit D / L-argenine	77	Mixed	First episode	Mixed	On completion	Ralph 2010—diagnostic CXR scoring system	% lung affected + 40 if cavitation seen. Total score 140.	Papuans: Median score 6/140, Range 2–15 Non papuans: Median score 12.5/140, Range 4–20.5	2/5

* Minimal- mild—lung fibrosis <50% of RUL, no change in architecture/ clouding or lung marking or vasculature. Moderate—lung fibrosis >50% of RUL, no change in architecture/ clouding or lung marking or vasculature / lung collapse / tortuous airways / bronchiectasis. Advanced—fibrosis of whole RUL, combined with collapse, bronchiectasis and tortuous airways

^†^Minimal—slight to moderate density not containing cavitation with total extent not exceeding lung volume on one side above the chondro-sternal junction. Extensive—slight to moderate density extending more than total volume of one lung or equivalent in both lungs

^‡^Study included patients treated for pulmonary, pleural or mediastinal TB—no disaggregated data available, so all included here

^¶^Minimal—Unilateral or bilateral. Lesions of slight to moderate density with no cavitation. Involvement should not exceed space above 2nd chondrosternal junction and the spine of the 4th or body of 5th vertebra. Moderate—Unilateral or bilateral. Disseminated lesions of slight-moderate density may extend through total volume of 1 lung or equivalent in both lungs. Dense/confluent lesions limited to 1/3 of one lung. Total diameter of cavitation must be <4cm. Advanced—more extensive than moderate.

^#^Grade I—minimal change in 1 zone, with no cavities. Grade II—2–3 zones involved, or 1 zone with cavitation. Grade III—severe involvment of >3zones, with or without cavitation

**Results stratified according to the presence of Chronic Airway Obstruction (CAO) on spirometry, as defined by a ratio of the post-bronchodilator forced expiratory volume in 1 second (FEV_1_) to forced vital capacity (FVC) ratio <0.7, and the % predicted FEV_1_< lower limit of normal: patients with CAO (n = 24), without CAO (n = 46). Mean and standard deviation for time since treatment and radiology findings given.

The prevalence of cavitation was higher in studies of re-treatment patients (68.8–83.7%), and those treated for MDR disease (51.5–69.7%), compared to those with fully sensitive, mixed, or unspecified sensitivities (8.3–49.7%). Only 1 study performed repeat imaging; this demonstrated a reduction in the prevalence of cavitation during the 6-month follow-up period from the end of TB treatment, but the findings were limited by a small sample size and 25% loss to follow-up[34]

### Pleural disease

The only radiological feature consistently reported in studies of pleural TB sequelae was the presence of residual pleural thickening, but the thoracic area covered by this thickening was not routinely reported. Residual thickening >10mm was seen in 19.6–46.0% of patients in 4 studies (n = 223) (Table 5). One study reported both CXR and CT findings following pleural TB, with mild pleural thickening >2mm seen in 50.0% (18/36) on CXR, and 60.0% (21/35) on CT[32].

**Table 5:** Studies of residual pleural thickening (RPT) on completion of treatment for TB pleural effusion.

Imaging modality	Author, Year	Country	Study design	Number of participants	Participant HIV status	Prevalence of pathology (%)	Quality score
CXR	Kunter, 2002[60]	Turkey	Retrospective cohort	47	Not specified	RPT>2mm: 63.8% RPT>10mm: 25.5%	2/5
CXR	Uskal, 2005[61]	Turkey	Retrospective cohort	121	Not specified	RPT>2mm: 52.1%	3/5
CXR	Wong, 2005[62]	Hong Kong	Retrospective cohort	70	Not specified	RPT>10mm: 41.4%	2/5
CXR	Barbas, 1991[63]	Brazil	Prospective cohort	44	Not specified	RPT>2mm: 52.3%	2/5
CXR	de Pablo, 1997 [64]	Spain	Prospective cohort	56	Mixed	RPT>2mm: 42.9% RPT>10mm: 19.6%	3/5
CXR	Frye, 1997[65]	America	Retrospective cohort	20	Positive	Any RPT: 65.0%	3/5
CXR	Galarza, 1995 [33]	Spain	RCT—steroid use*	60	Negative	Any RPT: 8.3%	2/5
CXR	Wyser, 1996 [32]	South Africa	RCT—steroid use*	36	Negative	RPT>2mm: 50.0%	4/5
CT	Wyser, 1996 [32]	South Africa	RCT—steroid use*	35	Negative	RPT>2mm: 60.0%	4/5
CT	Seiscento, 2007 [66]	Brazil	Prospective cohort	50	Not specified	RPT>10mm: 46.0%	3/5

*Date from study arm including steroids excluded

### Relationship of imaging changes and other respiratory parameters

Although several studies described spirometry following PTB disease, only 2 studies directly related physiological impairment to imaging findings. The first showed a statistically significant inverse correlation between both FEV_1_ (forced expiratory flow in 1-second) and FVC (forced vital capacity), and the extent of radiographic abnormality on CXR in 127 adults who were a median of 11 months (IQR 6–18 months) post completion of TB treatment[39]. The second described imaging findings in patients with (n = 24) and without (n = 46) fixed airway obstruction on spirometry following treatment completion. Those with airway obstruction had had more previous episodes of TB (1.9+/-0.7 vs. 1.4+/-0.6, p = 0.009), but had more fibrocavitatory changes evident on CXR imaging[59].

The only study to relate imaging findings to functional capacity included 18 patients completing treatment for MDR-TB, and found a higher level of impairment amongst those with more marked radiographic damage: 64% of those with Grade I damage (7/11) failed to reach an expected 6-minute walking distance, compared to 100% of patients with Grade III damage (3/3). However, the findings of this study were limited by a small sample size and a lack of statistical testing[37]. Only one study described the relationship between imaging findings and patient quality of life. This was a cross-sectional study of 198 patients who had been treated for TB a mean of 16.5 years previously, and found no statistically significant difference in the symptoms, activity, impact, or overall St George’s Respiratory Questionnaire scores between those with pathology affecting ≤2 vs. >2 CXR zones[47].

## Discussion

This review suggests a high prevalence of residual structural lung pathology following PTB treatment, and highlights the contribution that CT imaging may make to our understanding of this pathology. Several gaps in the literature have been identified, including a paucity of prospective data on the evolution of post-TB lung damage over time, limited geographical coverage of studies, little data from HIV-infected adults, and little information on the relationship between structural pathology, physiological abnormalities, and patient centered outcomes.

Despite the increasing focus on long-term patient outcomes within the post-2015 TB agenda, international targets for the management of PTB disease remain microbiologically and mortality driven: standard short course treatment for fully sensitive disease is provided for 6-months with discharge from health services on completion and no routine follow-up thereafter. Thoracic imaging is not routinely performed at the conclusion of treatment, and national TB treatment programmes are required to report data on treatment completion and survival only. Our review describes a high prevalence of residual abnormalities on imaging following medical treatment for PTB, in keeping with existing evidence on the high burden of abnormal airway physiology following PTB[15, 21]. This is particularly high amongst patients treated for MDR disease, which is of concern given the increasing prevalence of drug resistance in many high TB prevalence settings[67]. Our review suggests that there may be a substantial burden of undetected lung damage amongst patients completing TB treatment, but that our understanding of the combined structural/physiological nature of this damage, and the consequences of this for patients is limited. There remains insufficient evidence on which to base any changes to imaging and follow-up practices within existing TB programs.

Our review demonstrates differences in the prevalence and patterns of lung damage detected using CXR and CT imaging. CXR studies focused on cavitation, bronchiectasis, and fibrosis, and reported cavitation as the most common finding. In CT studies the prevalence of bronchiectasis was higher than that of cavitation, with possible features of ongoing inflammation (nodules and consolidation), emphysema, and mosaicism which may reflect small airways disease also reported. Whilst access to CT imaging is unlikely to be available in LMICs for routine assessment of patients after PTB treatment, limited use within research studies may have a role in accurately phenotyping disease, and identifying pathologies that could otherwise be missed. Studies performing paired CXR and CT imaging after pulmonary TB will then be required to determine the sensitivity of CXR imaging used within National Treatment Programs in LMICS for this CT defined structural damage.

Efforts to better define post-TB lung pathology will need to include an understanding of its evolution over time if we are to improve long-term patient outcomes. We identified a paucity of prospective data on the trajectory of imaging defined post-TB lung disease. This limitation is also seen in our understanding of the evolution of lung function following PTB, which is based on cross-sectional studies[68]. Uncertainty therefore remains about the rate and drivers of recovery/deterioration in those with post-TB lung damage. Factors influencing the progression of lung damage may include modifiable exposures such as smoking, biomass fuel[69, 70], and ongoing respiratory tract infections[71]. Further research in this area may inform strategies to prevent decline in this group.

Few studies attempted to examine the relationship between lung damage and patient functional capacity, symptom burden, and quality of life. The available data suggest that exercise capacity may decrease with increasing extents of structural pathology, but sample sizes in the one study to assess this were low[37]. The only study relating structural pathology to quality of life was vulnerable to selection bias, and the nature of this relationship therefore remains unclear[47]. A cohort study of patients with extensively destroyed lung after TB disease has confirmed a high incidence of infective exacerbations and mortality in this group[72], but further prospective data are required on outcomes amongst those with residual respiratory damage of a broader range of patterns and severity, from the time of treatment.

We identified a paucity of data from sSA and from HIV-positive patients. Given the convergence of a high TB prevalence and multiple risk factors for disease progression in sSA, data from this region is critical to our understanding of the public health impact of residual lung damage following TB treatment. HIV-positive individuals have a higher risk of developing TB disease, so should also be a focus of research. Although this group have atypical PTB presentations, with less structural damage seen at diagnosis[73], they may experience a differential pattern of progression of lung disease during or after treatment due to the effects of opportunistic infections, immune reconstitution syndrome and HIV-related immune activation on the lung[67, 74–76]. HIV-positive patients may also be particularly vulnerable to decreased quality of life and impaired functional capacity related to residual damage, and further analysis of respiratory outcomes in this group is urgently required[77].

The quality of studies identified in this review varied substantially. None included imaging prior to TB diagnosis, thus the presence of structural damage present prior to TB disease was not truly excluded. The majority of studies failed to specify how the radiological abnormalities reported on imaging were defined, and did not use gold-standard methods of reporting, making them vulnerable to misclassification of outcomes. Selection bias was a common issue; many studies were unclear about the reference population from which participants were drawn. The cross-sectional studies imaging patients sometime after treatment completion consistently struggled to locate eligible patients, and were limited by survival bias.

The strengths of our review include the use of several databases with no limitation on study dates, and the use of reference and citation review to identify additional papers. The study is limited by the inclusion of English language articles only. The heterogeneity of patient populations, treatment regimens, and the timing and modality of imaging meant that we were unable to perform a meta-analysis.

## Conclusion

This systematic review identified a high burden of structural pathology after PTB treatment. A better understanding of the nature of this pathology, evolution of disease over time, factors driving deterioration, and the impact of respiratory pathology on patients’ lives and livelihoods is needed to guide clinical management and health-service planning. Data on the sensitivity of CXR and spirometric indices for structural changes seen on CT imaging are required, together with studies examining post-TB lung damage in HIV-infected individuals and those from sSA. Further investigation of these areas is required if we are to meet the post-2015 TB aim of reducing the long-term detrimental impact of TB disease on patients’ lives and livelihoods.

## Supporting Information

S1 File Systematic Review Protocol. (DOCX)

S2 File PRISMA Checklist. (DOC)
