# Supplementary material for: A Systematic Review of the Prevalence and Pattern of Imaging Defined Post-TB Lung Disease
Source: PLoS One. 2016 Aug 12;11(8):e0161176. doi: 10.1371/journal.pone.0161176 (PMC4982669; doi:10.1371/journal.pone.0161176)
Supplement: S1 File — (DOCX) [file pone.0161176.s001.docx]

PROTOCOL: A Systematic Review of the Prevalence and Pattern of Imaging Defined Post-TB Lung Disease

Prospero registration

CRD42015027958

Authors

Dr J Meghji Malawi Liverpool Wellcome Clinical Research Programme

Liverpool School of Tropical Medicine

Malawi Liverpool Wellcome Trust

Queen Elizabeth Central Hospital

PO Box 30096, Chichiri, Blantyre 3. Malawi.

jamilah.meghji@lstmed.ac.uk

H Simpson Liverpool School of Tropical Medicine

hopesimpson@hotmail.co.uk

Prof SB Squire Liverpool School of Tropical Medicine

bertie.squire@lstmed.ac.uk

Dr K Mortimer Liverpool School of Tropical Medicine

kevin.mortimer@lstmed.ac.uk

Contributions

JM conceived of the idea of the systematic review, and designed the protocol.

JM and HS performed the review and extracted the data.

JM drafted the article, which was reviewed by HS and KM.

Both KM and BS provided senior supervision for the systematic review and manuscript authorship.

All authors have approved the final version of the manuscript.

JM is the guarantor of the content of the article.

Funding

JM is funded by a Wellcome Trust Clinical PhD Fellowship (106065/Z/14/A).

The protocol was designed and review conducted independently of the funding institution.

1. **INTRODUCTION**

**Rationale**

WHO targets for the management of pulmonary tuberculosis (PTB) disease are microbiologically driven. Standard short course treatment for fully sensitive disease is provided for 6 months with discharge from health services on completion and no routine follow-up, and tuberculosis trial outcomes focus on mortality and microbiological cure with little emphasis on morbidity. However, it is well known that pulmonary tuberculosis leads to chronic respiratory damage. Residual obstructive, restrictive, and mixed lung function deficits are seen (1-5), and structural changes including bronchiectasis, parenchymal destruction, cavitation, and fibrosis have been documented after treatment completion (6-8). Persistent symptoms include chronic cough and breathlessness (9-12).

The definition of lung disease is complex: spirometry, imaging, and symptom burden capture different dimensions of disease. The correlation between each of these features, and their association with morbidity / mortality varies between respiratory pathologies, but it has been recognised that severity indices which capture multiple dimensions of disease are best able to predict patient outome in conditions such as bronchiectasis and chronic obstructive airways disease (13-15). However, many studies estimating the prevalence of post-TB lung disease (PTLD) focus on spirometric deficits only, and relatively few have estimated the prevalence of residual structural damage on imaging. In addition to capturing a different dimension of respiratory disease, and perhaps therefore different patterns of respiratory disease, chest imaging allows us to understand the pathology underlying abnormal respiratory function in a more precise manner than spirometry.

Understanding the prevalence and pattern of imaging defined post-TB lung disease is therefore crucial to further studies investigating the long-term outcomes of patients with such residual damage after PTB treatment.

**Research question**

What is the prevalence and pattern of imaging defined (CT or CXR) respiratory pathology in adults, following treatment for pulmonary TB disease?

Population: HIV infected and uninfected adult patients

Excluded – patients with Pulmonary TB in context of non-HIV related immunosuppression (Eg. chemotherapy, TNFa therapy, malignancy, transplantation)

Interventions: Pulmonary, pleural, or miliary TB disease

Medically treated with antimicrobial agents

Excluded - additional treatments given (Eg. Steroids, immunomodulation, surgery) in addition to antimicrobial agents

Comparator: N/a

Outcomes: Chest radiograph or computerised tomography scan performed at or after TB treatment completion.

Imaging findings reported using severity scores or % prevalence of abnormal features.

1. **METHODS**

**Information sources**

Articles to be included:

- Published literature only
- No limits on publication date
- English language articles
- Cross-sectional, cohort, randomised control trials in which consecutive cases recruited

Electronic searches are to be conducted in Medline, Pubmed, Scopus, Web of Science, and The Cochrane Library, and are to be supplemented by:

- Reference reviews
- Citation searching
- Handsearching key journals / thesis / conference proceedings / trial registries

Date of search: July 2016

**Electronic literature search strategy**

The search terms used were designed to capture articles referring to the following: Pulmonary tuberculosis AND (CXR imaging OR CT imaging).

1. Medline search

| Category | Criteria | Search terms |
| --- | --- | --- |
| Population | Pulmonary Tuberculosis | “Tuberculosis, Pulmonary”[Mesh] OR  “tuberculosis, miliary[Mesh] OR  “tuberculosis, pleural”[Mesh}  OR  "pulmonary TB"OR "pulmonary tuberculosis" |
| Outcome | CXR Imaging | “thoracic radiography"[MeSH]  OR  “chest x-ray" OR "chest radiograph" OR "CXR" |
| Outcome | CT imaging | “computed tomography”[MeSH]  OR  "CT" OR "comput* tomography" |

1. Pubmed search

| Category | Criteria | Search terms |
| --- | --- | --- |
| Population | Pulmonary Tuberculosis | “Tuberculosis, Pulmonary”[Mesh] OR  “tuberculosis, military[Mesh] OR  “tuberculosis, pleural”[Mesh}  OR  "pulmonary TB"OR "pulmonary tuberculosis" |
| Outcome | CXR Imaging | “radiography, thoracic"[MeSH]  OR  “chest x-ray" OR "chest radiograph*" OR "CXR" |
| Outcome | CT imaging | “tomography, x-ray computed”[MeSH]  OR  "CT" OR "comput* tomography" |

*((((((("chest radiograph*") OR CXR) OR "chest x-ray") OR "radiography, thoracic"[MeSH])) OR ((("comput* tomography") OR CT) OR "Tomography, X-Ray Computed"[MeSH]))) AND ((((("tuberculosis, pulmonary"[MeSH]) OR "tuberculosis, pleural"[MeSH]) OR "tuberculosis, miliary"[MeSH]) OR "pulmonary tuberculosis") OR "pulmonary TB")*

1. Scopus search

| Category | Criteria | Search terms |
| --- | --- | --- |
| Population | Pulmonary Tuberculosis | "pulmonary tuberculosis" OR  “pleural tuberculosis” OR  “miliary tuberculosis” |
| Outcome | CXR Imaging | “chest x-ray" OR  "chest radiograph" OR  "CXR" |
| Outcome | CT imaging | "CT" OR  "comput* tomography" |

*( ( TITLE-ABS-KEY (****"chest x-ray"****) )  OR  ( TITLE-ABS-KEY (****"chest radiograph*"****) )  OR  ( TITLE-ABS-KEY (****"CXR"****) )  OR  ( TITLE-ABS-KEY (****"CT"****) )  OR  ( TITLE-ABS-KEY (****"Comput* tomography"****) ) )  AND  ( ( TITLE-ABS-KEY (****"pulmonary tuberculosis"****) )  OR  ( TITLE-ABS-KEY (****"pleural tuberculosis"****) )  OR  ( TITLE-ABS-KEY (****"miliary tuberculosis"****) ) )*

1. Web of science search

| Category | Criteria | Search terms |
| --- | --- | --- |
| Population | Pulmonary Tuberculosis | "pulmonary tuberculosis" OR  “pleural tuberculosis” OR  “miliary tuberculosis” |
| Outcome | CXR Imaging | “chest x-ray" OR  "chest radiograph" OR  "CXR" |
| Outcome | CT imaging | "CT" OR  "comput* tomography" |

1. Cochrane database search

| Category | Criteria | Search terms |
| --- | --- | --- |
| Population | Pulmonary Tuberculosis | "pulmonary tuberculosis" |

1. **STUDY RECORDS**

Data management

References to be managed in Endnote 7

Study selection

1. Duplicates to be removed in Endnote
2. Title and abstracts of remaining articles to be reviewed by 2 independent readers (JM and HS), and included if felt to be relevant / in the event of uncertainty. All articles identified by either reader to be included for full text review.
3. Full text for papers identified to be reviewed by 2 independent readers (JM and HS), and eligibility formally assessed.
4. Disagreements on inclusion / exclusion to be resolved by discussion.
5. Review by a 3^rd^ reader (BS) to be requested if required.

Data extraction

1. Data extraction to be performed independently into an excel spreadsheet independently by 2 readers (JM & HS).
2. Disagreements to be resolved by discussion.
3. Review by a 3^rd^ reader to be requested if required.

Data items

Data to be extracted for the following variables:

Study characteristics:

- Author
- Year of publication
- Country
- Study design
- Study dates (month/year)

Patient characteristics:

- Pattern of TB disease – pleural, pulmonary, miliary
- First episode / retreatment
- Microbiological evidence for disease
- Drug sensitivity of organism
- HIV status of participants
- Treatment outcome
- Participant age
- Other inclusion / exclusion criteria used

TB treatment received

- Duration of treatment
- Treatment regimen

Imaging findings

- Timing of imaging, post treatment completion
- Imaging modality – CXR / CT imaging
- Number of imaging readers
- Definitions of imaging findings used
- % prevalence of the following: overall normal / abnormal imaging, cavitation, bronchiectasis, fibrosis, lung destruction, pleural thickening, other
- Severity scores used
- Severity score results

Associated factors

- Patient centred outcomes measured (Symptoms, health related quality of life, functional capacity)
- Lung function measured
- Relationship of associated factors to imaging described

1. **STUDY OUTCOMES**

Outcomes and prioritisation

Primary outcome: Prevalence of abnormal imaging findings (cavitation, bronchiectasis, fibrosis, pleural thickening)

Secondary outcomes: Prevalence of other abnormal imaging findings

Severity scoring systems, and results on imaging

Relationship between imaging findings and patient-centred outcomes / spirometry

Assessment of bias

Methodological quality to be judged using a modified version of the Newcastle-Ottowa score, in which the maximum score for a cohort study is 5 and for a cross-sectional study is 4.

Findings are to be reported in the review write up, and potential impact on conclusions discussed.

| Title | Explanation | Score |
| --- | --- | --- |
| Representativeness of cohort | - Truly representative of the average TB patient in the community - Somewhat representative of the average TB patient in the community - Selected groups - No description of the derivation of the cohort | 1 |
| Assessment of Exposure* | - Secure record - Structured interview - Written self report - No description | 1 |
| Demonstration that Outcome∞ not present at start | - Imaging prior to TB diagnosis - Statement of exclusion of those with known preceding lung disease | 1 |
| Assessment of Outcome∞ | - Independent blind assessment by 2 readers - Definitions of imaging findings (severity score and individual features) given | 1 |
| Adequacy of follow up of cohorts | - Complete follow up - Subjects lost unlikely to introduce bias - <20% of those starting treatment & surviving - Follow up rate <80% of those who survive - No statement | 1 |

*Exposure – pleural, pulmonary or miliary TB disease

∞Outcome – structural lung damage

1. **DATA SYNTHESIS**

Description

A description of data is to be provided.

If studies are felt to be sufficiently homogeneous, quantitative synthesis is to be performed and a summary estimate / pooled prevalence of each of the three main imaging findings expected (cavitation, fibrosis, bronchiectasis) will be reported.

Potential subgroups for analysis

1. HIV-infected vs. HIV-uninfected
2. Fully sensitive vs. MDR disease
3. Microbiologically proven (smear, GXP or culture) vs. unproven disease
4. Imaging within 1yr vs. after 1 year from treatment completion
5. First episode vs. retreatment
6. **REFERENCES**

1. Allwood BW, Myer L, Bateman ED. A systematic review of the association between pulmonary tuberculosis and the development of chronic airflow obstruction in adults. Respiration. 2013;86(1):76-85.

2. Ehrlich RI, Adams S, Baatjies R, Jeebhay MF. Chronic airflow obstruction and respiratory symptoms following tuberculosis: a review of South African studies. Int J Tuberc Lung Dis. 2011;15(7):886-91.

3. Menezes AM, Hallal PC, Perez-Padilla R, Jardim JR, Muino A, Lopez MV, et al. Tuberculosis and airflow obstruction: evidence from the PLATINO study in Latin America. Eur Respir J. 2007;30(6):1180-5.

4. Pasipanodya JG, Miller TL, Vecino M, Munguia G, Garmon R, Bae S, et al. Pulmonary impairment after tuberculosis. Chest. 2007;131(6):1817-24.

5. Hnidzo E SH, Churchyard G. Chronic pulmonary function impairment caused by initial and recurrent pulmonary tuberculosis following treatment. Thorax. 2000;55:32-8.

6. Long R MB, Dhar A, Manfresa J, Hershfield E, Athonisen N. Pulmonary tuberculosis treated with Directly Observed Therapy: Serial changes in lung structure and function. Chest. 1998;1998(113):933-43.

7. Lee JJ, Chong PY, Lin CB, Hsu AH, Lee CC. High resolution chest CT in patients with pulmonary tuberculosis: characteristic findings before and after antituberculous therapy. Eur J Radiol. 2008;67(1):100-4.

8. Al-Hajjaj MS, Joharjy IA. Predictors of radiological sequelae of pulmonary tuberculosis. Acta Radiol. 2000;41(6):533-7.

9. Baez-Saldana R, Lopez-Arteaga Y, Bizarron-Muro A, Ferreira-Guerrero E, Ferreyra-Reyes L, Delgado-Sanchez G, et al. A novel scoring system to measure radiographic abnormalities and related spirometric values in cured pulmonary tuberculosis. PloS One. 2013;8(11):e78926.

10. Ralph AP, Kenangalem E, Waramori G, Pontororing GJ, Sandjaja, Tjitra E, et al. High morbidity during treatment and residual pulmonary disability in pulmonary tuberculosis: under-recognised phenomena. PloS One. 2013;8(11):e80302.

11. M. Muniyandi RR, R. Balasubramanian, C. Nirupa, P. G. Gopi, K. Jaggarajamma, F. Sheela, P. R. Narayanan. Evaluation of post-treatment health-related quality of life (HRQoL) among tuberculosis patients. Int J Tuberc Lung Dis. 2007.

12. Page ID OJ, Onyachi N, Opira C, Odongo-Aginya E, Mockridge A, Byrne G, Denning DW. Chronic pulmonary aspergillosis complicating treated pulmonary tuberculosis in Gulu, Uganda. The Union, World Conference on Lung Health; Paris, France 2013 [Abstract]

13. Chalmers JD, Goeminne P, Aliberti S, McDonnell MJ, Lonni S, Davidson J, et al. The bronchiectasis severity index. An international derivation and validation study. Am J Respir Crit Care Med. 2014;189(5):576-85.

14. Celli BR, Cote CG, Marin JM, Casanova C, Montes de Oca M, Mendez RA, et al. The body-mass index, airflow obstruction, dyspnea, and exercise capacity index in chronic obstructive pulmonary disease. NEJM. 2004;350(10):1005-12.

15. Puhan MA, Garcia-Aymerich J, Frey M, ter Riet G, Anto JM, Agusti AG, et al. Expansion of the prognostic assessment of patients with chronic obstructive pulmonary disease: the updated BODE index and the ADO index. Lancet. 2009;374(9691):704-11.
